# Electroacupuncture activated local sympathetic noradrenergic signaling to relieve synovitis and referred pain behaviors in knee osteoarthritis rats

**DOI:** 10.3389/fnmol.2023.1069965

**Published:** 2023-03-07

**Authors:** Wei Chen, Xiao-Ning Zhang, Yang-Shuai Su, Xiao-Yu Wang, Heng-Cong Li, Yi-Han Liu, Hong-Ye Wan, Zheng-Yang Qu, Xiang-Hong Jing, Wei He

**Affiliations:** Institute of Acupuncture and Moxibustion, China Academy of Chinese Medical Sciences, Beijing, China

**Keywords:** knee osteoarthritis, electroacupuncture, synovitis, macrophage, sympathetic noradrenergic signaling, CXCL1, IL-6

## Abstract

**Introduction:**

Recent research has focused on the local control of articular inflammation through neuronal stimulation to avoid the systemic side effects of conventional pharmacological therapies. Electroacupuncture (EA) has been proven to be useful for inflammation suppressing and pain reduction in knee osteoarthritis (KOA) patients, yet its mechanism remains unclear.

**Methods:**

In the present study, the KOA model was established using the intra-articular injection of sodium monoiodoacetate (MIA) (1 mg/50 μL) into the knee cavity. EA was delivered at the ipsilateral ST36-GB34 acupoints. Hind paw weight-bearing and withdrawl thresholds were measured. On day 9, the histology, dep enrichment proteins, cytokines contents, immune cell population of the synovial membrane of the affected limbs were measured using HE staining, Masson staining, DIA quantitative proteomic analysis, flow cytometry, immunofluorescence staining, ELISA, and Western Blot. The ultrastructure of the saphenous nerve of the affected limb was observed using transmission electron microscopy on the 14th day after modeling.

**Results:**

The result demonstrated that EA intervention during the midterm phase of the articular inflammation alleviated inflammatory pain behaviors and cartilage damage, but not during the early phase. Mid-term EA suppressed the levels of proinflammatory cytokines TNF-α, IL-1β, and IL-6 in the synovium on day 9 after MIA by elevating the level of sympathetic neurotransmitters Norepinephrine (NE) in the synovium but not systemic NE or systemic adrenaline. Selective blocking of the sympathetic function (6-OHDA) and β2-adrenergic receptor (ICI 118,551) prevented the anti-inflammatory effects of EA. EA-induced increment of the NE in the synovium inhibited the CXCL1-CXCR2 dependent overexpression of IL-6 in the synovial macrophages in a β2-adrenergic receptor (AR)-mediated manner.

**Discussion:**

These results revealed that EA activated sympathetic noradrenergic signaling to control local inflammation in KOA rats and contributed to the development of novel therapeutic neurostimulation strategies for inflammatory diseases.

## 1. Introduction

Knee osteoarthritis (KOA) is a multifactorial joint disease, including joint degeneration, intermittent inflammation, and peripheral neuropathy. Biological anti-rheumatic drug therapies are expensive and likely to increase the risk of systemic immunosuppression, infections, and malignancies. Recent experimental efforts have focused on the local control of articular inflammation to avoid the systemic side effects of conventional pharmacological therapies. Neuronal stimulation, such as vagal stimulation, is an emerging field for regulating organ function and reestablishing physiological homeostasis during illness, in which the regulation of the immune system of the nervous system has been widely studied (Ulloa et al., 2017).

Although the precise mechanisms and processes of KOA pathogenesis are not yet fully understood, cartilage extracellular matrix fragments are observed to induce synovial inflammation by activating resident fibroblasts or macrophages, which in turn release pro-inflammatory cytokines, such as tumor necrosis factor-α (TNF-α), interleukin-1β (IL-1β), IL-6, IL-8, and other inflammatory mediators, and further recruit other immune cells, resulting in thickening of the synovial lining layer and synovial hyperplasia (Jenei-Lanzl et al., 2019; Woodell-May and Sommerfeld, 2020). Furthermore, chemokine and its receptors are crucial players in the pathological processes of osteoarthritis (OA), which are well-recognized for their role in the migration of circulating immune cells into injury sites (Sallusto et al., 2000). In the synovial fluid of KOA patients, CCL3 produced in osteoarthritic knees can chemo-attract circulating monocytes to the inflamed synovium through CCR1 (Zhao et al., 2020). In human synovial fibroblasts of OA, chemokine (CXC motif) ligand 1 (CXCL1) contributes to IL-6 expression through the CXCR2, c-Raf, MAPK, and AP-1 pathways (Hou et al., 2020). Macrophages are crucial mediators of OA-related synovial inflammatory activity and cartilage and bone pathologic responses (Griffin and Scanzello, 2019). The number of activated macrophages in knee joints correlated with the severity and symptoms of radiographic OA (Kraus et al., 2016). Depletion of macrophages in OA synovial explants substantially reduced the production of numerous cytokines, including TNF-α, IL-1β, IL-6, and IL-8 (Bondeson et al., 2010).

This low-level inflammation in the joint is believed to contribute to the development of peripheral sensitization and nociceptive pain (Krustev et al., 2015). Synovitis and baseline synovial thickening increase the rate of OA progression and are associated with increased pain and dysfunction in OA patients (Scanzello et al., 2011; Roemer et al., 2016). Moreover, attenuation of early-phase inflammation prevents pain and nerve damage in OA rats (Philpott et al., 2017). Therefore, we must find a strategy for suppressing synovitis to prevent peripheral sensitization and nociceptive pain in KOA patients.

The knee joint is mainly innervated by sensory and sympathetic nerves. Changes in peripheral joint innervation are supposed to be partly responsible for degenerative alterations in joint tissues and the development of OA (Grässel and Muschter, 2017). The loss of sympathetic nerve fibers was observed in the synovial tissue of patients with rheumatoid arthritis (Miller et al., 2000). As an integrative interface between the brain and the immune system, the sympathetic nerve plays a crucial role in inflammation control (Elenkov et al., 2000). The activation of sympathetic function-mediated baroreflex reduced joint inflammatory response in conscious rats (Bassi et al., 2015). A local sympathetic-immune pathway has been demonstrated to regulate arthritic joint inflammation through vagal nerve stimulation (Bassi et al., 2017). LC and RVLM, as the important sympathetic supraspinal centers, also participated in the anti-inflammatory effect in Parkinson’s disease, zymosan-induced inflammatory air pouch, and articular inflammation (Yoon et al., 2007; Neill and Harkin, 2018).

EA has been proven to be useful for pain reduction and inflammation suppression in KOA patients (Cherkin et al., 2009; Li et al., 2018; Liu et al., 2021). However, it is unclear whether the sympathetic function mediates the effect of EA on suppressing synovitis and alleviating referred hind paw pain in KOA rats. In the present study, we demonstrated the effect of EA on suppressing synovitis and referred pain, and its mechanism related to the activation of sympathetic function in MIA-induced KOA rats. We found that EA at the mid-term phase of inflammatory pain suppressed inflammation in the synovium on day 9 after MIA by activating sympathetic function in KOA rats. The EA-induced increase in sympathetic neurotransmitters (NEs) inhibited CXCL1-induced recruitment of circulating monocytes and accumulation of macrophages in synovial tissue, which over-expressed IL-6, in a β2AR-dependent manner. Our study provides evidence that EA can suppress synovitis to relieve inflammatory pain in KOA rats.

## 2. Materials and methods

### 2.1. Animals

Healthy adult male Sprague Dawley rats, weighing 180–200 g, were obtained from the National Institutes for Food and Drug Control (medical laboratory animal certificate number: SCXK (JING)2017–0005) and were permitted to acclimate to their environment for at least 1 week. Standard laboratory chow and water were provided *ad libitum*. The animals were kept in an ambient room with the following conditions: temperature (22 ± 4°C), humidity 60–65%, and light (7:00–19:00). General anesthesia was induced under 4% isoflurane and maintained under 2% isoflurane.

### 2.2. Intra-articular injection

The animals were deeply anesthetized (2–4% isoflurane; 100% oxygen at 1 l/min) until the cessation of all sensory reflexes. The right knee joint was shaved and swabbed with 100% ethanol. MIA (1 mg/ 50 μL dissolved in sterile saline), 6-OHDA (150 μg/ 50 μL, dissolved in a saline solution containing 0.02% of ascorbic acid; Deng et al., 2016), and ICI 118,551 (1.5 μg/ 50 μL, dissolved in sterile saline) were injected into the joint space, as previously described (Teixeira et al., 2020). The knee was then manually extended and flexed for 30 s to disperse the solution throughout the joint. The right knee of the control rats received sterile saline.

### 2.3. Electroacupuncture

For EA intervention, one acupuncture needle was inserted into the ipsilateral ST36 acupoint, and another needle was inserted into the ipsilateral GB34 acupoint. ST36 is located posterolateral to the knees and approximately 5 mm below the fibular head. GB34 is located in the depression anterior and inferior to the fibula head, 5 mm from the upper and outer sides of ST36. The selected EA parameters were 1 mA, dense-sparse wave (2/15 Hz), and 30 min per time.

### 2.4. Pain behavioral tests

**Figure 1:**
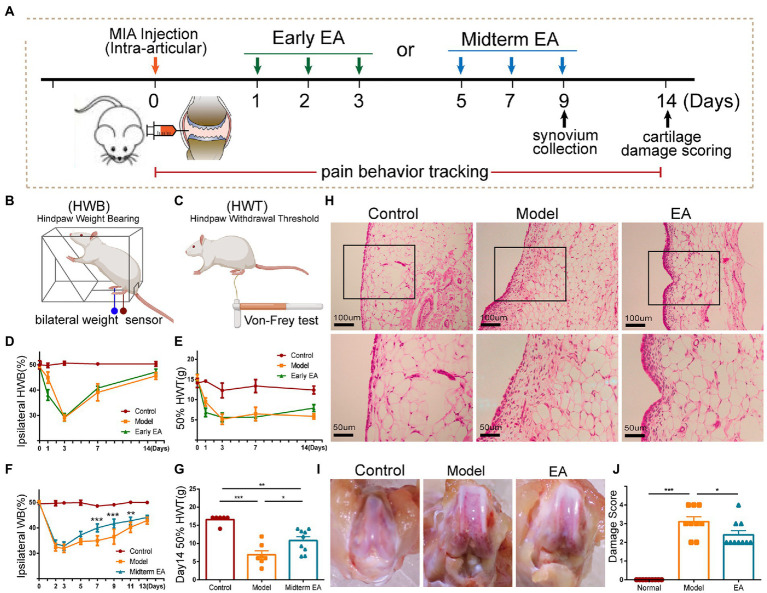
Midterm EA intervention alleviated the inflammatory pain behaviors in MIA-induced KOA rats. **(A)** The scheme on early EA and midterm EA. The percentage of ipsilateral weight bearing (WB) **(B,D)** and the 50% hind paw withdrawal threshold (HPWT; **C,E**) in the model group and the early EA group was decreased from day 1 to day 14 after MIA injection in comparison with that of the control group, yet no significant difference was observed between the model group and the early EA group from day 3 to day 14 after MIA injection (*n* = 6, *p* > 0.05). On day 1 after MIA injection, the percentage of ipsilateral WB and the 50% HPWT were even decreased in the early EA group in comparison with the model group. *p < 0.05, **p < 0.01 vs. control. **(F)** The percentage of ipsilateral WB was increased in the midterm EA group on day 7 and day 9 after the MIA injection in comparison with the model group (*n* = 10, *p < 0.05, **p < 0.01, ***p < 0.001). **(G)** The ipsilateral 50% HPWT in the midterm EA group was increased on day 14 after the MIA injection in comparison with that of the model group (*n* = 10, *p < 0.05, **p < 0.01, ***p < 0.001). **(H)** On day 9 after MIA injection, hyperplasia and inflammatory cell infiltration of the synovium occurred in the model group, which was alleviated in the midterm EA group. **(I)** On day 14 after the MIA injection, cartilage damage occurred in the model group, which was alleviated in the midterm EA group (*n* = 3). **(J)** The cartilage damage score in the midterm EA group was decreased in comparison with that in the model group on day 14 after the MIA injection (*n* = 9, *p < 0.05, **p < 0.01, ***p < 0.001).

The weight-bearing deficit (WBD) was measured as a sign of spontaneous pain (Bove et al., 2003). All rats were acclimated to the test environment for 30 min. The animals were placed in a perspex chamber (model BIO-DWB-AUTO-R; Bioseb, Boulogne, France) to place their hind limbs on an independent force plate (Figure 1B). The weight borne on the ipsilateral hind paw was calculated as a percentage of the total weight borne on the hind limbs. Each value is the mean of three replicates.

The tactile hypersensitivity of the plantar of the hind paw was measured using von Frey hairs as a sign of secondary allodynia (Muley et al., 2016). The rats were placed in a Plexiglas chamber and allowed to acclimate for approximately 30 min, or until their exploratory behavior ceased. The withdrawal mechanical threshold of the ipsilateral hind paw was assessed using a modification of the Dixon up-down method (Figure 1C; Chaplan et al., 1994). The 50% withdrawal threshold was determined using the following formula: 50% threshold = (10[Xf + κδ])/10,000; where Xf = value (in log units) of the final von Frey hair used; k = tabular value for the pattern of positive/negative responses, and δ = mean difference (in log units) between stimuli.

### 2.5. Macroscopic scoring of the articular cartilage lesion

The rats were sacrificed on day 14 after intra-articular injection of MIA. A photograph of knee cartilage was taken with a digital camera (Canon). Two independent observers scored cartilage damage on the knee cartilage surface in a blinded manner (Takeshita et al., 2011), with severity increasing from 0 to 4: 0 = normal appearance, 1 = rough surface, 2 = moderate lesion on the cartilage surface, 3 = severe lesion of subchondral bone, 4 = appearances of osteophyte formation with the severe lesion of subchondral bone (Janusz et al., 2001). The mean score was determined for each group.

### 2.6. Histopathology of synovial tissue

Anesthetized rats were fixed with saline and 4% paraformaldehyde through transcardial perfusion. Following perfusion–fixation, the collected synovial tissues were post-fixed in 4% paraformaldehyde and then embedded in paraffin wax. To assess the severity of inflammation and structural changes in the synovial tissue, 5-μm-thick sagittal sections of the synovial tissue were prepared and stained with hematoxylin and eosin. The severity of arthritis in the joint was observed according to the intensity of the lining layer hyperplasia and inflammatory cell infiltration, as described previously (Liao et al., 2020).

### 2.7. Biochemical analysis of blood and synovium

Whole blood was collected from animals through cardiac puncture for hemogram analysis. To obtain serum, whole blood was allowed to clot in a polypropylene tube at room temperature for 120 min. The tubes were centrifuged at 2000 × g for 20 min. To obtain plasma, we collected the blood in an EDTA anticoagulant tube and centrifuged it at 1600 × g for 15 min at 4°C for 30 min. The supernatant serum and plasma were absorbed, packaged separately, and stored at −80°C for testing. The right synovium of the knee joint was homogenized for the quantitative determination of the NE and cytokine levels.

Cytokine levels of CXCL1, IFN-γ, TNF-α, IL-1β, IL-6, IL4, IL-10, and IL-13 in the serum and synovium were measured using the MSD MULTI-SPOT Assay System (Proinflammatory Panel 2 (rat), N05059A-1; Meso Scale Discovery, United States). The levels of plasma NE (KA1877, Abnova, Taiwan), epinephrine (KA1877, Abnova, Taiwan), and CORT (KGE009, R&D Systems, Minnesota, United States) were measured using enzyme immunoassay (EIA) or ELISA kits. The NE levels in the synovium were measured using the ELISA kit (BA E-5200, BioTNT, Germany) according to the manufacturer’s instructions.

### 2.8. Immunofluorescence staining

Anesthetized rats were fixed with saline and 4% paraformaldehyde *via* transcardial perfusion. Following perfusion–fixation, the collected synovial tissues were post-fixed in 4% paraformaldehyde, then embedded in an artificial medium (Shandon Cryomatrix™, 120 mL, Thermo Fisher Scientific, United States), frozen, and cut into 20-μm sections on a cryostat (Thermo Fisher Scientific, Microm International FSE, Germany). After an initial wash in 0.1 M PBS (pH 7.4), the tissues were preincubated in a solution of 3% normal goat and 0.5% Triton X-100 in 0.1 M phosphate-buffered solutions (PB, pH 7.4) for 30 min to block non-specific binding. The sections were then incubated with primary antibodies (Anti-TH: Ab112; Anti-C-Fos: Ab208942; Anti-iNOS, Ab15323; Anti-Dopamine beta Hydroxylase: Ab19353; Anti-CRF, Ab8901; Abcam, England) for 24 h at 4°C, followed by goat/donkey anti-rabbit Alexa Fluor 488 secondary antibody or donkey anti-mouse/rabbit/sheep Alexa Fluor 488/594 secondary antibody (1:500; Molecular Probes) for 2 h at room temperature. The tissues were then counterstained with blue fluorescent DAPI nuclei acid (1/40000, D3571, 10 mg, Invitrogen, United States) for 5 min to label cell nuclei. After a final wash in 0.1 M PBS, the slides were covered and slipped with PBS–glycerol. Negative controls were performed by omitting the primary antibodies during the staining procedure. The slides were observed with a confocal imaging system (FV1200, Olympus, Japan) and analyzed using the Olympus Image Processing Software by an investigator who was blinded to the group. Approximately 20 randomized sections from each group were analyzed. All immunohistochemistry for each staining combination was performed simultaneously to ensure staining consistency.

### 2.9. Western blot analysis

Standard western blot techniques were performed on the knee synovium protein sample. Incubation processed with primary antibody (ADRB2A, YT5048,1:500; ADRA1A YT0354,1:500; ADRA2A YT0298,1:500; p-Erk1/2 #9101,1:1000; Erk1/2 #4695,1:1000; MEK #8727,1:1000; p-MEK1/2 #3958,1:1000; GAPDH #2118/#97,166,1:2000, CST) (CXCR2, bs-4836R,1:1000; β-actin, bs-0061R 1:4000, bioss) was diluted in 1X TBST overnight. We diluted the secondary antibody (HRP-conjugated Affinipure Goat Anti-Rabbit IgG (H + L), 1:1000, SA-00001-2, Proteintech HRP-conjugated Affinipure Goat Anti-Mouse IgG (H + L), 1:1000, SA-00001-1, Proteintech) in 1X TBST and incubated the membrane for 1 h at room temperature or on a shaker. Protein bands were detected with ECL substrate (34,080, Thermo Fisher Scientific) through an imaging system (ChemiDoc Touch V3, BioRad) and analyzed using ImageJ software.

### 2.10. Flow cytometry

Knee synovium tissues dissected from rats were processed to prepare cell suspensions using the Skeletal Muscle Dissociation Kit (130–098-305, Miltenyi Biotec). The cell surface was stained with phycoerythrin (PE) anti-rat CD86 and FITC anti-rat CD11b (BD Bioscience, United States). After incubation, flow cytometric analysis was performed using flow cytometry (FACS Celesta, BD Bioscience). The data were further analyzed using the FlowJo software.

### 2.11. DIA quantitative proteomic analysis

Synovial tissue was collected as previously described. Proteomic analysis was conducted at Shanghai Applied Protein Technology Co., Ltd. The synovial tissues of Model group rats (n = 8) and EA group rats (*n* = 8) were homogenized using an MP FastPrep-24 homogenizer (24 × 2, 6.0 m/s, 60 s, twice), and then SDT buffer (100 mM DTT, 4% SDS, 150 mM Tris–HCl, pH 8.0) was added. The lysates were further sonicated and boiled for 15 min. After centrifugation at 14000 × g for 40 min, the supernatant was quantified with the BCA Protein Assay Kit (Bio-Rad, United States). The aliquots of each sample were mixed into a single sample for quality control and the construction of a data-independent acquisition (DDA) library. Protein digestion was implemented following the filter-aided sample preparation procedure (Shen et al., 2021). DDA analysis was performed with a high-performance liquid chromatography system Easy nLC-1,200 (Thermo Scientific). Buffer A:0.1% formic acid (FA, 06450, Fluka); buffer B:0.1% FA, 84% Acetonitrile (ACN, I592230123, Merck). The column was balanced with 95% buffer A. The peptide was first loaded onto an EASY-Spray TM C18 Trap column (Thermo Fisher Scientific, P/N 164946, 3 μM, 75 μM × 2 cm) and then separated on an EASY-Spray TM C18 LC Analytical Column (Thermo Fisher Scientific, ES802, 2 μM, 75 μM × 25 cm) with a linear gradient of buffer B at a flow rate of 250 nl/min over 90 min. The liquid separation gradient of buffer B is as follows: 0–40 min, the linear gradient is from 8 to 30%; 40–50 min, the linear gradient is from 30 to 100%; 50–65 min, the linear gradient increases to 100% and is maintained. The separated samples were analyzed using the Q-Exactive HFX mass spectrometer (Thermo Fisher Scientific). MS detection method was positive ion, the scan range was 300–1800 m/z, MS1 scan resolution was 60,000 at 200 m/z, the target of automatic gain control (AGC) was 3e6, and the maximum injection time (IT) was 50 ms. Each complete MS–SIM scan was preceded by 20 MS2 scans. The resolution of the MS2 scan was 30,000, the AGC target was 3e6, the maximum IT was 120 ms, and the normalized collision energy was 27 eV. DDA data were directly imported into the Spectronaut software (SpectronautTM 14.4.200727.47784) to construct a spectral library. The database can be downloaded from http://www.uniprot.org. All reported data were based on a protein identification confidence of 99%, as defined through a false discovery rate (FDR) of ≤1%. Each sample was mixed with 2 μg of the iRT standard peptide, and DIA mass spectrometry was performed. Each DIA cycle contained one complete MS–SIM scan, and 30 DIA scans covered a mass range of 350–1,650 m/z with the following settings: SIM full scan resolution was 60,000 at 200 m/z; AGC: 3e6; maximum IT: 50 ms. DIA scans were set at a resolution of 30,000; AGC: 3e6; maximum IT: auto; normalized collision energy was 30 eV. Spectronaut was used to search the pre-existing spectral library against DIA data for analysis. All results were filtered based on FDR < 1%. We used the Wu Kong platform^1^ for missing-value imputation. Differentially expressed proteins (DEPs) between two groups were selected with a fold change (FC) greater than 1.2 or lower than 0.83 and a *p*-value less than 0.05 (Student’s *t*-test). They were annotated with GO, and further bioinformatic analysis was performed.

### 2.12. Transmission electron microscopy

A segment of the saphenous nerve was isolated proximal to the right knee joint, placed in 4% glutaraldehyde diluted with (0.1 M sodium cacodylate buffer) and stored at 4°C for at least 1 week. The nerve samples were then removed from the fixative and rinsed three times with 0.1 M sodium cacodylate buffer. The samples were fixed in 1% osmium tetroxide for 2 h, rinsed with PBS (3 times, 5 min), and then placed in 0.25% uranyl acetate (4°C) overnight. The samples were then dehydrated in a graduated series of acetone: 50, 70, and 90% for 15 min separately, and finally 100% for 10 min. The samples were then dried in 100% acetone for 10 min. The samples were infiltrated with Epon–Araldite resin and placed in a 2:1 ratio of dried 100% acetone to resin for 2 h, followed by a 1:1 ratio of dried 100% acetone to resin for 2 h. The samples were then placed in 100% Epon–Araldite resin for 2 h and cured in an oven at 36°C for 12 h, 48°C for 12 h, and 60°C for 24 h. Finally, using an LKB Huxley ultramicrotome with a diamond knife, the samples were sectioned into 70-nm thick slices. Cross-sectional slices of nerves were placed into a copper wire grid consisting of 300 individual squares per inch (each square measuring 83 × 3 × 58 mm) and then stained with 2% aqueous uranyl acetate for 10 min, followed by lead citrate for 4 min.

### 2.13. Statistical analysis

All data are expressed as mean ± standard error of the mean. Statistical analysis was conducted using GraphPad Prism 6 (GraphPad Prism Software Inc., San Diego, United States). Normality was checked for all analyses. Two-way ANOVA followed by Tukey’s multiple comparison tests was performed to evaluate EA’s effects on weight-bearing deficits (WBD) and hind paw pain thresholds. For subsequent experiments, a one-way ANOVA was performed followed by Tukey’s multiple comparison test. A *p*-value <0.05 was considered statistically significant.

## 3. Results

### 3.1. Electroacupuncture at the midterm phase of inflammatory pain alleviated inflammatory pain behaviors and cartilage damage in monoiodoacetate-induced knee osteoarthritis rats

First, we observed the effect of EA during the early phase of the inflammatory pain (abbreviated as early EA), which is performed daily from day 1 to day 3 after MIA injection (Figures 1A–E). In MIA-induced KOA rats, inflammatory pain behaviors, such as the ipsilateral weight bearing deficit (WBD) and the decrease in the hind paw withdrawal threshold (HPWT), began on day 1 after MIA injection, reached the worst on day 3 after MIA injection, and then began to recover in the following days (Figures 1D,E). Therefore, the initial 3 days of the inflammatory pain were considered to be its early phase. However, early EA did not alleviate the WBD and HPWT at any time point of the intervention, which even worsened on day 1 after the MIA injection (Figures 1D,E). These results indicated that early EA is not feasible to alleviate inflammatory pain behaviors in KOA rats.

EA at the midterm phase of the inflammatory pain (abbreviated as midterm EA) was performed every other day from day 5 to day 9 after the MIA injection (Figures 1A,F,G). Midterm EA alleviated the WBD at day 7 and day 9 after MIA injection, whereas there was no significant WBD difference between the model and EA groups at other time points of the intervention (Figure 1F). WBD is a measure of spontaneous pain associated with joint inflammation (Lorton and Bellinger, 2015). We then observed the histopathological alterations of the synovium and found that midterm EA suppressed the hyperplasia of the synovium on day 9 after MIA injection (Figure 1H). The HPWT was used to determine secondary allodynia distal to the injured joint that can be indicative of nerve injury (Malfait, 1999). On day 14 after the MIA injection, the HPWT increased, and the cartilage damage score was decreased in the midterm EA group in comparison with that in the model group (Figures 1G,I,J), implying that EA alleviated the secondary allodynia on day 14 after MIA injection. These findings indicate that midterm EA may be optical to alleviate the spontaneous pain behaviors, and hyperplasia of the synovial membrane on day 9 after MIA injection, and relieve the secondary allodynia and cartilage damage on day 14 after MIA injection in KOA rats.

### 3.2. Electroacupuncture regulates the inflammatory response in the synovium of knee osteoarthritis rats

**Figure 2:**
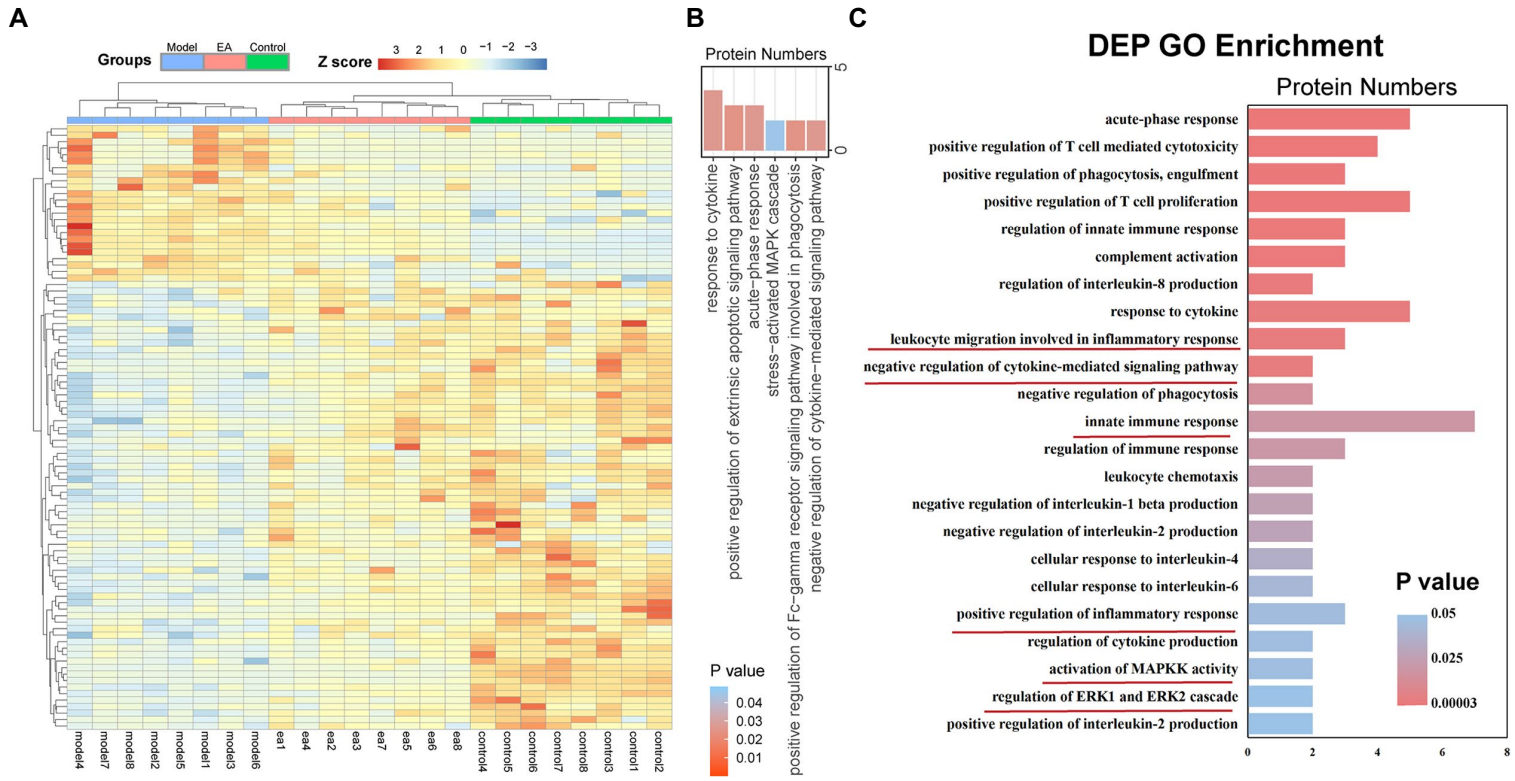
EA regulates the inflammatory response in the synovium of KOA rats. The features of differentially expressed proteins (DEPs) of the synovitis in the model group compared with those of the EA group. **(A)** DEP profiles were hierarchically clustered and shown as a heatmap wherein 222 proteins were up-regulated (red) or down-regulated (blue). **(B)** Gene ontology analysis of DEPs: Enriched with terms of response to cytokine, positive regulation of extrinsic apoptotic, signaling pathway, acute-phase response, stress−activated MAPK cascade, positive regulation of Fc-gamma receptor signaling pathway involved in phagocytosis, and negative regulation of cytokine−mediated signaling pathway. **(C)** Gene ontology analysis of DEPs: Enriched in terms related to immune regulation and response (*n* = 8): cellular response to IL-6, negative regulation of interleukin-1 beta production, negative regulation of cytokine-mediated signal pathway, the regulation of innate immune response, leukocyte migration involved in the inflammatory response, positive regulation of inflammatory response, activation of MAPKK activity, and regulation of ERK1 and ERK2 Cascade.

We detected the proteomics of the synovium of rats on the 9th day. We screened according to *p* < 0.05 and foldchange (FC) > 1.2. A total of 222 DEPs were identified, of which 144 were up-regulated and 78 were down-regulated (Figure 2A). GO analysis of these DEPs revealed that they were enriched in terms of response to cytokine, positive regulation of extrinsic apoptotic, signaling pathway, acute-phase response, stress−activated MAPK cascade, positive regulation of Fc-gamma receptor signaling pathway involved in phagocytosis, and negative regulation of cytokine−mediated signaling pathway (Figure 2B). We found that they were enriched in terms related to immune regulation and response. Thus, we analyzed the DEPs in the model group and the Ea group. DEPs were enriched in terms of cellular response to IL-6, negative regulation of interleukin-1 beta production, the production of cytokines and other regulatory factors. The remaining terms include the negative regulation of cytokine-mediated signal pathway, the regulation of innate immune response, leukocyte migration involved in the inflammatory response, positive regulation of inflammatory response, activation of MAPKK activity, and regulation of ERK1 and ERK2\Cascade (Figure 2C). These findings suggest that macrophage-secreted cytokines may be involved in the regulation of EA on synovitis.

### 3.3. Midterm electroacupuncture suppressed the levels of the proinflammatory cytokines in the synovium of monoiodoacetate-induced knee osteoarthritis rats

**Figure 3:**
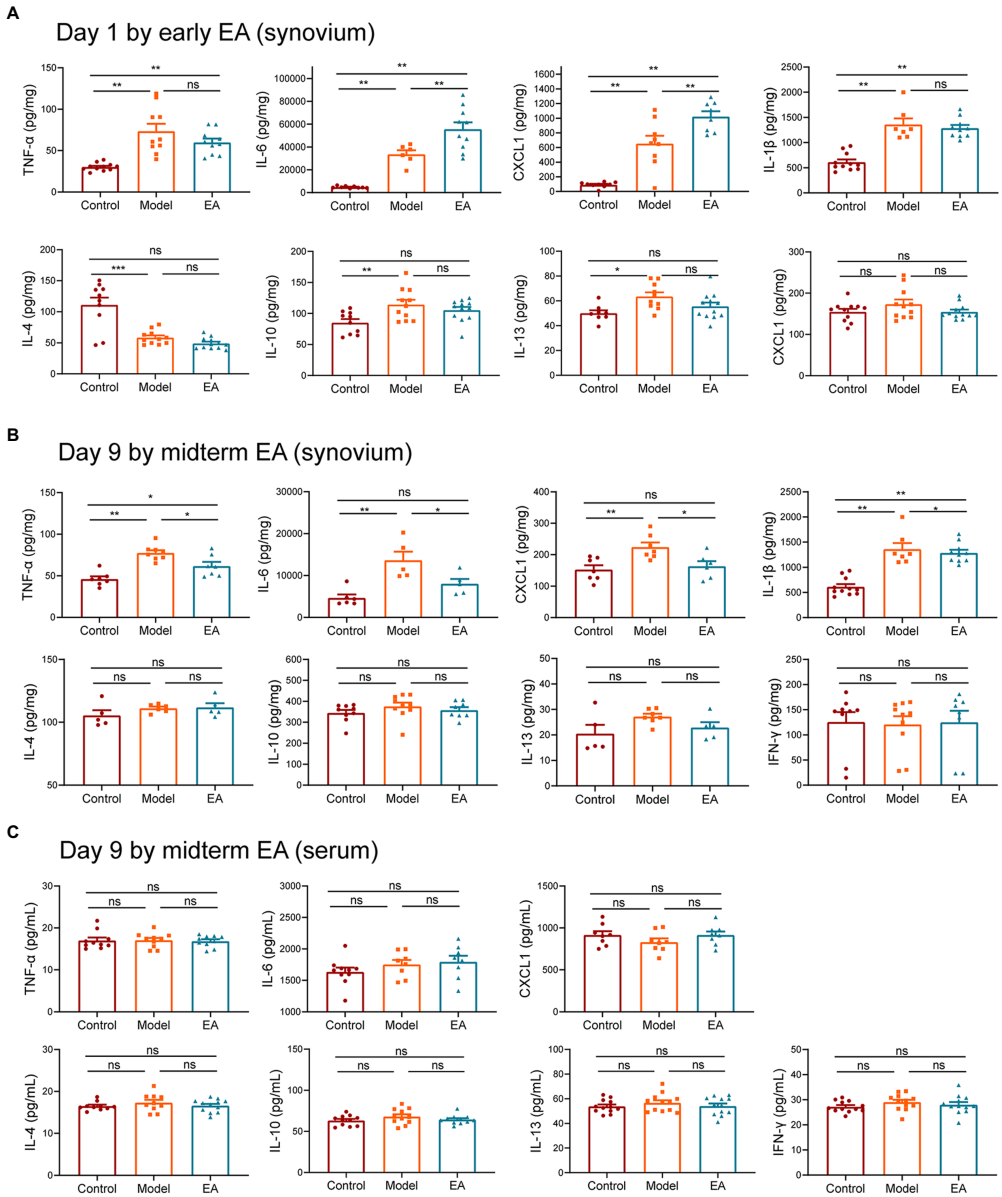
Midterm EA suppressed the levels of proinflammatory cytokines CXCL1, IL-6, TNF-α, and IL-1β in the synovium of MIA-induced KOA rats. **(A)** The levels of proinflammatory cytokine CXCL1, IL-6, TNF-α, and IL-1β were increased on day 1 after MIA injection; however, the levels of proinflammatory cytokine CXCL1, IL-6 in the early EA group were increased more than those in the model group, whereas no significant difference was observed in the levels of proinflammatory cytokine TNF-α and IL-1β between the model group and the early EA group on day 1 after MIA injection (*n* = 12, *p < 0.05, **p < 0.01, ***p < 0.001). **(B)** The levels of proinflammatory cytokine CXCL1, IL-6, TNF-α, and IL-1β were increased on day 9 after MIA injection in the model group, whereas they were decreased in the midterm EA group (*n* = 9, *p < 0.05, **p < 0.01, ***p < 0.001). No statistical difference was observed in the levels of cytokine IFN-γ, IL-4, IL-10, and IL-13 in the synovium among the three groups. **(C)** No statistical difference was observed in the levels of all cytokines in the serum among the three groups (*n* = 9, *p < 0.05, **p < 0.01, ***p < 0.001).

To uncover the molecular mechanisms underlying EA’s protective effects, we examined the cytokine levels in the synovium and serum. The results demonstrated that the levels of proinflammatory cytokines CXCL1, IL-6, TNF-α, and IL-1β were increased on day 1 after MIA injection; however, the levels of proinflammatory cytokines CXCL1 and IL-6 were increased more by early EA (Figure 3A), correlating with the behavioral results that early EA was unable to alleviate inflammatory pain behaviors in KOA rats. We then observed the change in cytokines by midterm EA on day 9 after the MIA injection. The results demonstrated that when compared with the control group, the levels of proinflammatory cytokines CXCL1, IL-6, TNF-α, and IL-1β were increased in the model group but decreased in the EA group (Figure 3B). The levels of proinflammatory cytokine IFN-γ and anti-inflammatory cytokines IL-4, IL-10, and IL-13 in the synovium did not differ significantly among the three groups (Figure 3B). Furthermore, the serum levels of all cytokines did not differ significantly among the three groups (Figure 3C). These findings suggest that midterm EA suppressed the levels of proinflammatory cytokines CXCL1, IL-6, TNF-α, and IL-1β in the synovium and that the inflammation in MIA-induced KOA rats is limited to the joint but not systemic.

### 3.4. Electroacupuncture-activated sympathetic noradrenergic signaling to suppress the levels of CXCL1 and IL-6 in the synovium in a β2AR-mediated manner

**Figure 4:**
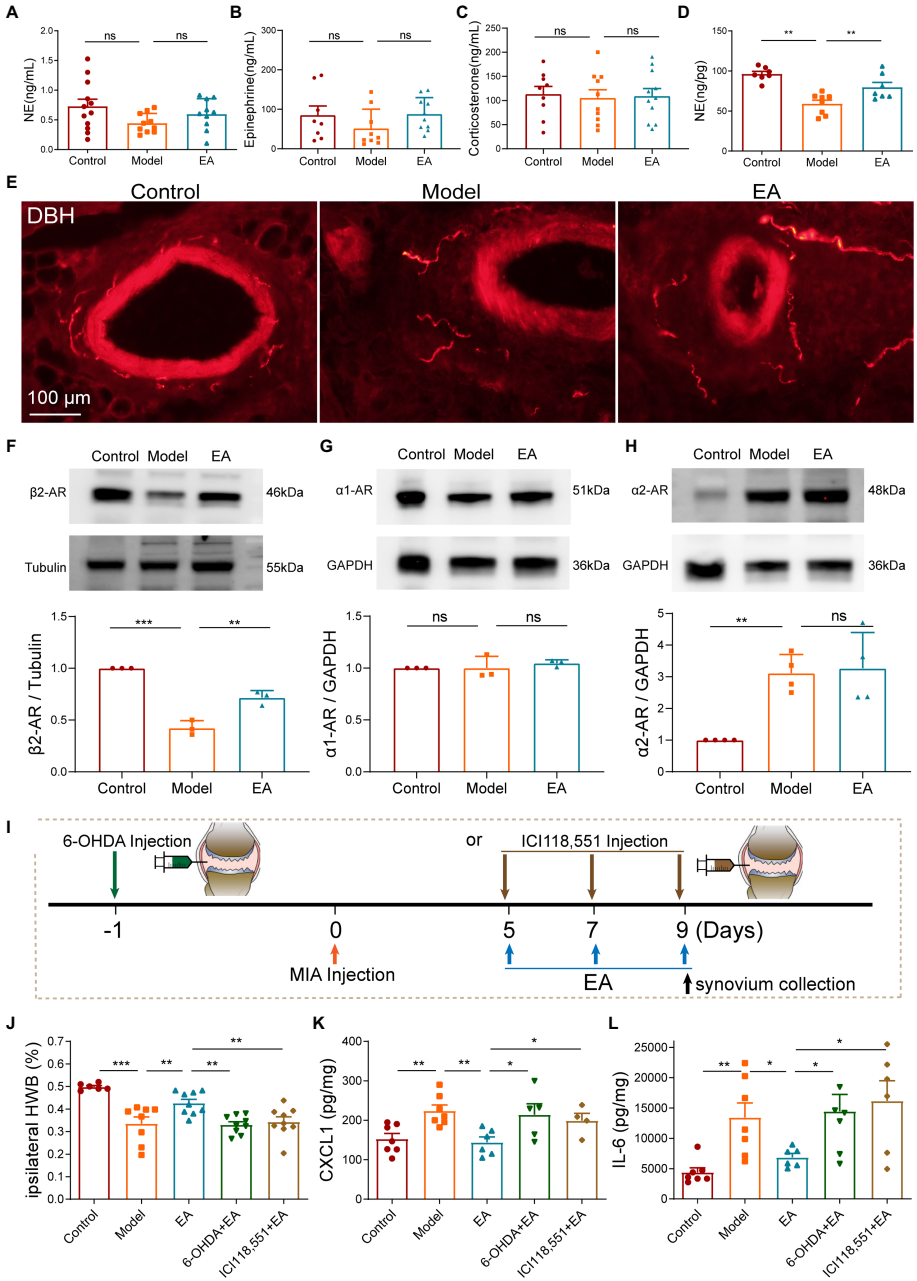
Midterm EA increased sympathetic nerve transmitters NE to suppress the levels of CXCL1 and IL-6 in the synovium in a β2AR-dependent manner on day 9 after MIA injection. **(A–C)** No significant difference was observed in the levels of NE, epinephrine, and corticosterone in the plasma among the three groups (*n* = 10–12, *p < 0.05, **p < 0.01, ***p < 0.001). **(D)** The levels of NE in the synovium were decreased in the model group in comparison with those of the control but were increased in the EA group (*n* = 10, *p < 0.05, **p < 0.01, ***p < 0.001). **(E)** Immunohistochemical staining of the synovium revealed that the expression of DBH-positive sympathetic nerve was decreased in the model group and was increased in the EA group (*n* = 3). **(F)** The expression of β2-AR in the synovium was decreased in the model group in comparison with the control but was increased in the EA group (*n* = 3, *p < 0.05, **p < 0.01, ***p < 0.001). **(G)** No significant difference was observed in the expression of α1-AR in the synovium among the three groups (*n* = 3, *p < 0.05, **p < 0.01, ***p < 0.001). **(H)** The expression of α2-AR in the synovium was increased in the model and EA groups, but there was no significant difference between that in the model group and the EA group (*n* = 3, *p < 0.05, **p < 0.01, ***p < 0.001). **(I)** Intra-articular injections of 6-OHDA and ICI 118,551 were performed to block neurotransmitters NE and its β2-AR. **(J)** Intra-articular injection of 6-OHDA and ICI 118,551 blocked the protective effect of EA on the WBD. **(K,L)** Intra-articular injection of 6-OHDA and ICI 118,551 blocked EA-induced suppression of the levels of CXCL1 and IL-6 (*n* = 8, *p < 0.05, **p < 0.01, ***p < 0.001).

To characterize the role of sympathetic function in the anti-inflammatory effect of midterm EA in MIA-induced KOA rats, we measured the levels of norepinephrine (NE), epinephrine, and corticosterone (CORT) in the plasma among the three groups but no significant differences were observed (Figures 4A–C). We examined the levels of norepinephrine in the synovium and found that the level of NE in the synovium decreased in the model group but increased after EA on day 9 after MIA injection (Figure 4D). Immunohistochemical staining of the synovium also revealed that the expression of dopamine-β-hydroxylase (DBH) positive sympathetic nerve was decreased in the model group and increased in the EA group (Figure 4E). These results indicated that the sympathetic nerve transmitters NE in the synovium played a role in the anti-inflammatory effect of EA. The expressions of α1, α2, and β2-AR in the synovium on day 9 after MIA injection were measured using Western Blot technology. We found that the expression of β2-AR in the synovium was decreased in the model group compared with the control group, but increased after EA treatment (Figure 4F). Furthermore, no significant difference was observed in the expression of α1-AR in the synovium among the three groups (Figure 4G). The expression of α2-AR in the synovium was increased in the model and EA groups, but there was no significant difference between that in the model group and the EA group (Figure 4H). To further identify whether NE and β2-AR participated in the anti-inflammatory effect of EA, we used chemical denervation of noradrenergic fibers (6-OHDA) and the β2-AR antagonists (ICI 118,551) to assess the ipsilateral WB and cytokine levels in the synovium of MIA-induced KOA rats (Figure 4I). The results demonstrated that intra-articular injection 6-OHDA and ICI 118,551 blocked the effect of EA on the WB deficits (Figure 4J). Moreover, 6-OHDA and ICI 118,551 eliminated EA-induced suppression of CXCL1 and IL-6 (Figures 4K,L). These results indicated that EA increased the level of NE to suppress the levels of CXCL1 and IL-6 in the synovium in a β2AR-mediated manner.

**Figure 5:**
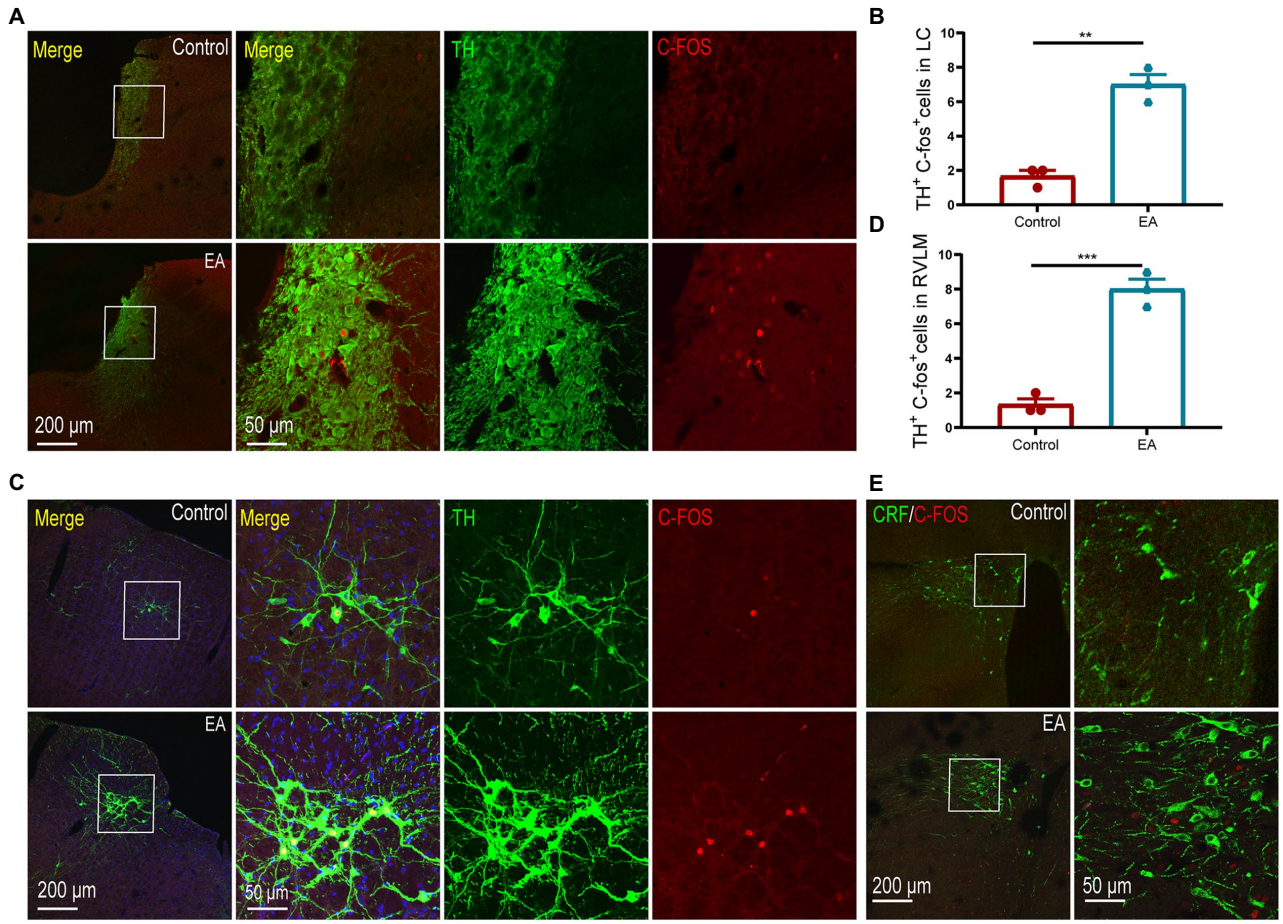
EA activated the neurons in the LC and RVLM. **(A,B)** TH + c-fos positive neurons in the LC increased after EA. **(C,D)** TH + c-fos positive neurons in the RVLM increased after EA. **p < 0.01. **(E)** No co-expression of CRF + c-fos positive neurons was observed in the PVN of rats in the two groups. *n* = 4 in each group.

As LC and RVLM are important sympathetic centers, we observed that EA activated noradrenergic neurons in the LC and RVLM, as indicated by the increase of c-fos + TH positive neurons in the LC, and the RVLM increased after EA (Figures 5A–D). The paraventricular nucleus of the hypothalamus (PVN) is involved in the activation of the sympathetic function. However, consistent with the results of plasma CORT levels, we did not observe activation of the c-fos + CRH positive neurons in the PVN (Figure 5E), indicating that the PVN did not participate in the anti-inflammatory effect of EA in KOA rats.

### 3.5. Electroacupuncture suppressed the CXCL1-CXCR2-dependent overexpression of IL-6 in macrophages of the synovium in knee osteoarthritis rats

**Figure 6:**
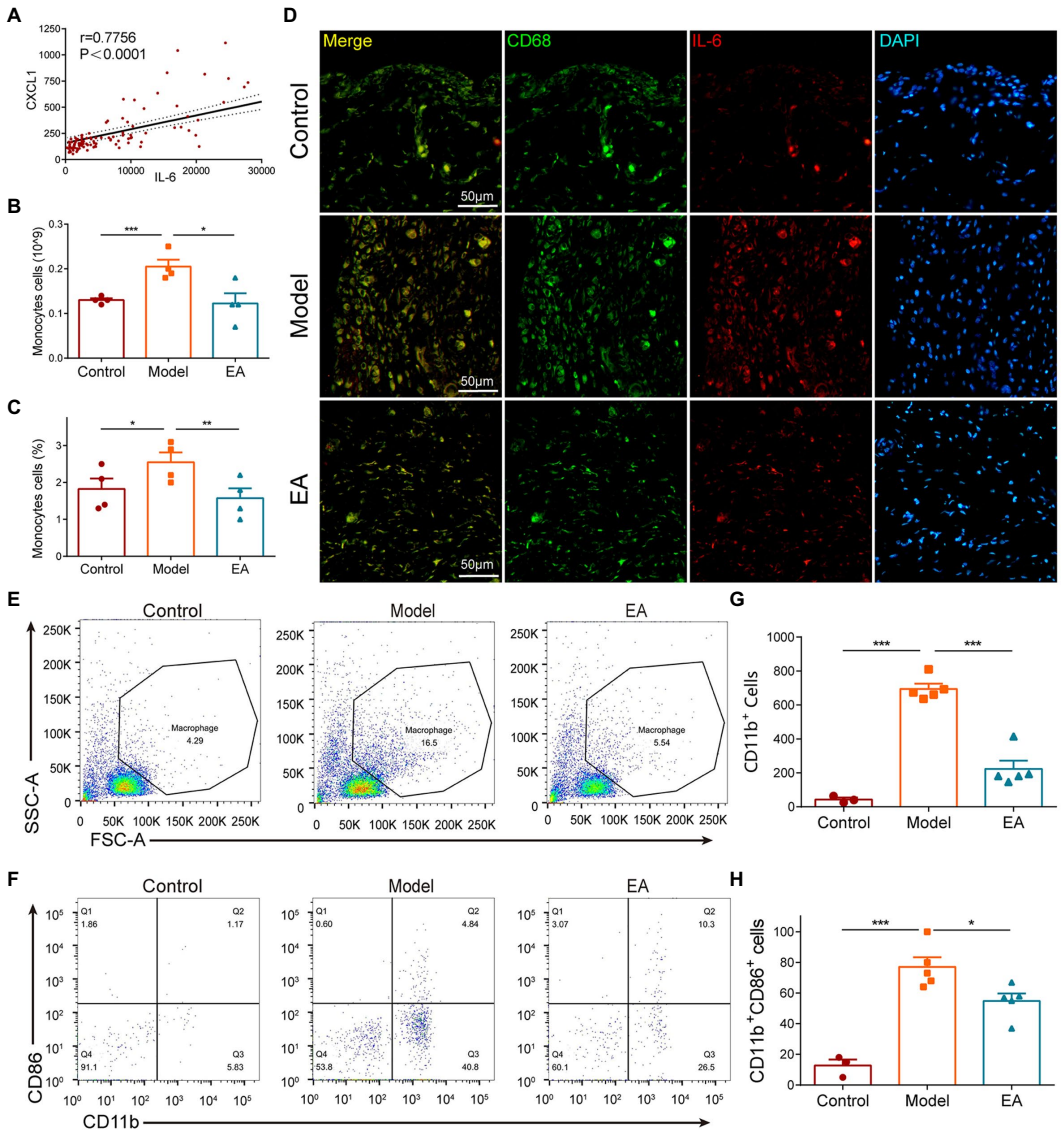
EA inhibited the CXCL1-dependent overexpression of IL-6 in macrophages of the synovium. **(A)** All groups except the control group exhibited a positive correlation between the levels of CXCL1 and IL-6 in the synovium (data not shown). **(B)** The number of circulating monocytes was increased in the model group but decreased in the EA group. **(C)** The percentage of circulating monocytes increased in the model group but decreased in the EA group. **(D)** The co-expression of IL-6 and CD68 + macrophage was increased in the synovium in the model group but decreased in the EA group. **(E–H)** In Flow Analysis, the number of CD11b + CD86 + M1 macrophages in the synovium was increased in the model group but decreased in the EA group. (*n* = 5, *p < 0.05, **p < 0.01, ***p < 0.001).

According to the aforementioned results, the chemokine CXCL1 and the cytokine IL-6 were the most important cytokines in the EA-induced anti-inflammatory effect. We performed a correlation analysis of the two cytokines and found that there was a positive correlation between the levels of CXCL1 and IL-6 in the synovium in all groups (Figure 6A) except for the control group (data not shown). The hemogram analysis demonstrated that the number and percentage of the circulating monocytes were increased in the model group but decreased in the EA group (Figures 6B,C). Immunohistochemical staining of the synovium also revealed that the co-expression of IL-6 and CD68 + macrophage was increased in the synovium in the model group, but decreased in the EA group (Figure 6D). Flow Analysis revealed that the number of CD11b + CD86 + M1 macrophages in the synovium was increased in the model group but decreased in the EA group (Figures 6E–H). These results indicated that EA inhibited the CXCL1-CXCR2-dependent overexpression of IL-6 of macrophages in the synovium of MIA-induced KOA rats.

**Figure 7:**
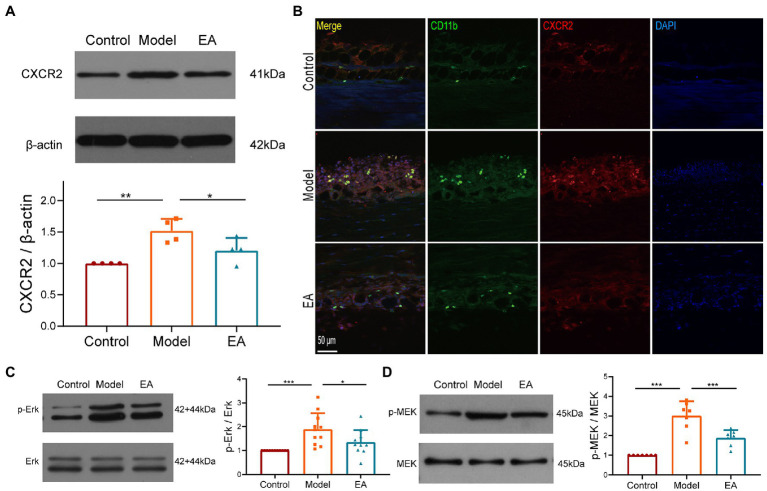
The anti-inflammatory effect of EA on synovitis is mediated by suppressing the CXCL1-CXCR2 axis. **(A)** The expression of CXCR2 protein in the synovium was measured using Western Blotting (*n* = 3, *p < 0.05, **p < 0.01). **(B)** The co-expression of CXCR2 and the CD11b + macrophage in the synovium (*n* = 3). **(C)** The expression of phosphorylation ERK in the synovium. **(D)** The expression of phosphorylation MEK in the synovium. *n* = 4–6 in each group (*n* = 3, *p < 0.05, **p < 0.01, ***p < 0.001).

We then examined the expression of CXCR2, the receptor of chemokine CXCL1, and observed that the expression of CXCR2 protein was increased in the synovium on day 9 after MIA but decreased after EA (Figure 7A). Immunochemical staining revealed that the CXCR2-positive cells were collated with the CD11b + macrophages, and EA suppressed the increase in CXCR2 expression in the synovium (Figure 7B). C-Raf is a typical signal transducer involved in chemokine receptor activation, including CXCR2, and the MAPK pathway comprises several key signaling components, including ERK. We found that EA also suppressed the phosphorylation of ERK and MEK in the synovium of KOA rats (Figures 7C,D). These findings imply that the anti-inflammatory effect of EA on the synovitis of MIA-induced-KOA rats is mediated by suppressing the CXCL1-CXCR2 axis through the MAPK/ERK signaling pathway.

### 3.6. Electroacupuncture alleviated peripheral nerve damage in monoiodoacetate-induced knee osteoarthritis rats

**Figure 8:**
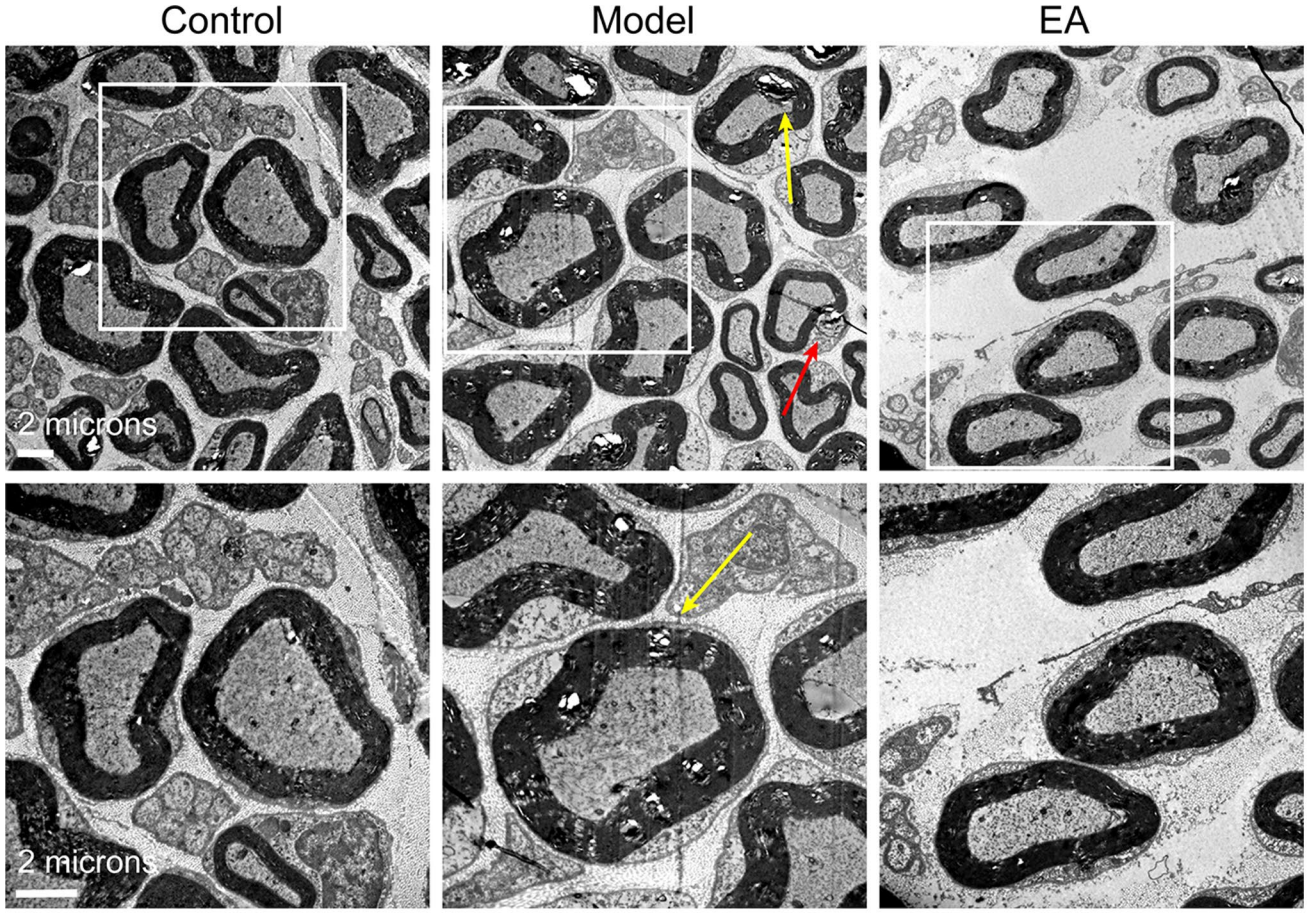
Electroacupuncture alleviated peripheral nerve damage and reduced firing rate in MIA-induced KOA rats. Representative sections of electron micrographs of axons found in the saphenous nerves were taken on day 14 after MIA in the control, model, and EA groups. The yellow arrow indicates nerve myelin damage; the red arrow indicates Schwann cell damage. The scale bar is 6 mm (*n* = 3).

Finally, we observed the peripheral nerve damage which is associated with the referred hind paw pain on day 14 after MIA in KOA rats. Saphenous nerve demyelination was observed on day 14 after MIA, yet EA prevented saphenous nerve demyelination (Figure 8A). These results suggested that peripheral nerve damage occurred on day 14 after MIA in KOA rats, but EA prevented peripheral nerve damage by inhibiting saphenous nerve demyelination.

## 4. Discussion

In the present study, midterm EA suppressed the onset of synovitis on day 9 after MIA injection in KOA rats by activating the sympathetic noradrenergic signaling pathway. EA stimulated the release of the sympathetic NE, which inhibited CXCL1-CXCR2-dependent overexpression of IL-6 in the synovial macrophages in a β2AR-mediated manner, and relieved referred hind paw pain on day 14 after MIA. Our study confirmed that EA suppressed CXCL1-CXCR2-dependent overexpression of IL-6 in the synovial macrophages to relieve referred hind paw pain by activating the sympathetic noradrenergic signaling in MIA-induced KOA rats. Moreover, the inflammation in MIA-induced KOA rats was limited to the joint but not the systemic level. EA only elevated the level of NE in the synovium but not systemic NE or systemic adrenaline, to suppress the levels of the proinflammatory cytokines CXCL1, IL-6, TNF-α and IL-1β in the synovium, thereby exerting a local anti-inflammatory effect.

In recent decades, the role of the sympathetic nervous system (SNS) and, in particular, its major peripheral catecholamine NE in the pathogenesis of OA has attracted growing interest. Understanding the SNS in inflammatory joint disease elucidated various pro and anti-inflammatory perspectives: the early neurogenic proinflammatory role and the immunosuppressive role in later stages (Elenkov et al., 2000). Therefore, the time point of intervention is crucial for controlling joint inflammation. In the present study, early EA (EA from day 1 to day 3 after MIA) neither suppressed the levels of the proinflammatory cytokines nor alleviated the referred hind paw pain, indicating that in the acute phase of inflammatory pain of the MIA-induced KOA rats, EA is ineffective for controlling the joint inflammation. We observed that midterm EA (EA from day 5 to day 13 after MIA) suppressed the levels of proinflammatory cytokine CXCL1, TNF-α, IL-1β, and IL-6 in the synovium, and alleviated the WBD on day 9 after MIA, suggesting that midterm EA is appropriate for suppressing inflammatory pain behaviors and synovitis.

Synovial cells express several ARs, such as α1-AR, α2-AR, and β2-AR, which can respond to varying NE levels (Sohn et al., 2021). The loss of sympathetic nerve fibers is speculated to be accompanied by decreased concentrations of NE, causing this neurotransmitter to bind predominantly to α-adrenoceptors (higher affinity) but not β2-AR (Sohn et al., 2021). The reduction of the NE level and the β2-adrenoceptor signaling in synovial tissues are involved in the inflammatory response of synoviocytes in adjuvant-induced arthritic rats (Wu et al., 2019). Furthermore, NE inhibits TNF-α and IL-8 secretion mediated by β2-ARs in synovial macrophages derived from OA and RA patients (Jenei-Lanzl, 2015). The β2-adrenergic agonist salbutamol has been identified as a potent suppressor of collagen-induced arthritis (Malfait et al., 1999). In the present study, we observed that EA reversed both the decrease in the concentration of NE and the expression of β2-AR after MIA, thereby suppressing the inflammation in the synovium. Despite the increased expression of α2-AR in the model group, no obvious difference was observed between the model group and the EA group. We conclude that EA increases NE levels to suppress synovitis by activating β2-AR.

Inflammatory conditions, such as those present in KOA synovial tissue, lead to the immigration of monocytes and their differentiation into macrophages. In MIA-induced KOA rats, damage-associated molecular patterns (DAMPs) trigger monocyte/macrophage recruitment and activation in the joints as drivers of OA symptoms and pathology (Griffin and Scanzello, 2019). In OA synovium and cartilage, cytokines associated with M1 macrophages (e.g., TNF-α, IL-1β, and IL-6) are well established for their role in stimulating pro-catabolic mediators, such as aggrecanases and MMPs. Mice with a pro-inflammatory M1 bias had developed greater synovial inflammation and cartilage pathology (Zhang et al., 2018). Accordingly, the focus on synovial macrophages as drivers of OA symptoms has led to therapeutic strategies involving the removal of synovial macrophages. Crosstalk between SNS and the immune system is crucial for health and well-being. β2-AR has been proposed to mediate the crosstalk between sympathetic neurons and immune cell trafficking (Lorton and Bellinger, 2015). Activating local colonic sympathetic innervations, but not systemic sympathetic activation attenuates colitis by limiting immune cell extravasation in an β2AR-mediated manner (Schiller et al., 2021). In the present study, EA inhibited circulating monocyte trafficking to synovial macrophages and decreased the levels of M1 macrophage-associated cytokines TNF-α, IL-1β, and IL-6 in an β2AR-mediated manner. These findings indicate that EA-induced inhibition of synovitis is mediated by activating sympathetic noradrenergic signaling to limit monocyte/macrophage cell extravasation in the synovium.

**Figure 9:**
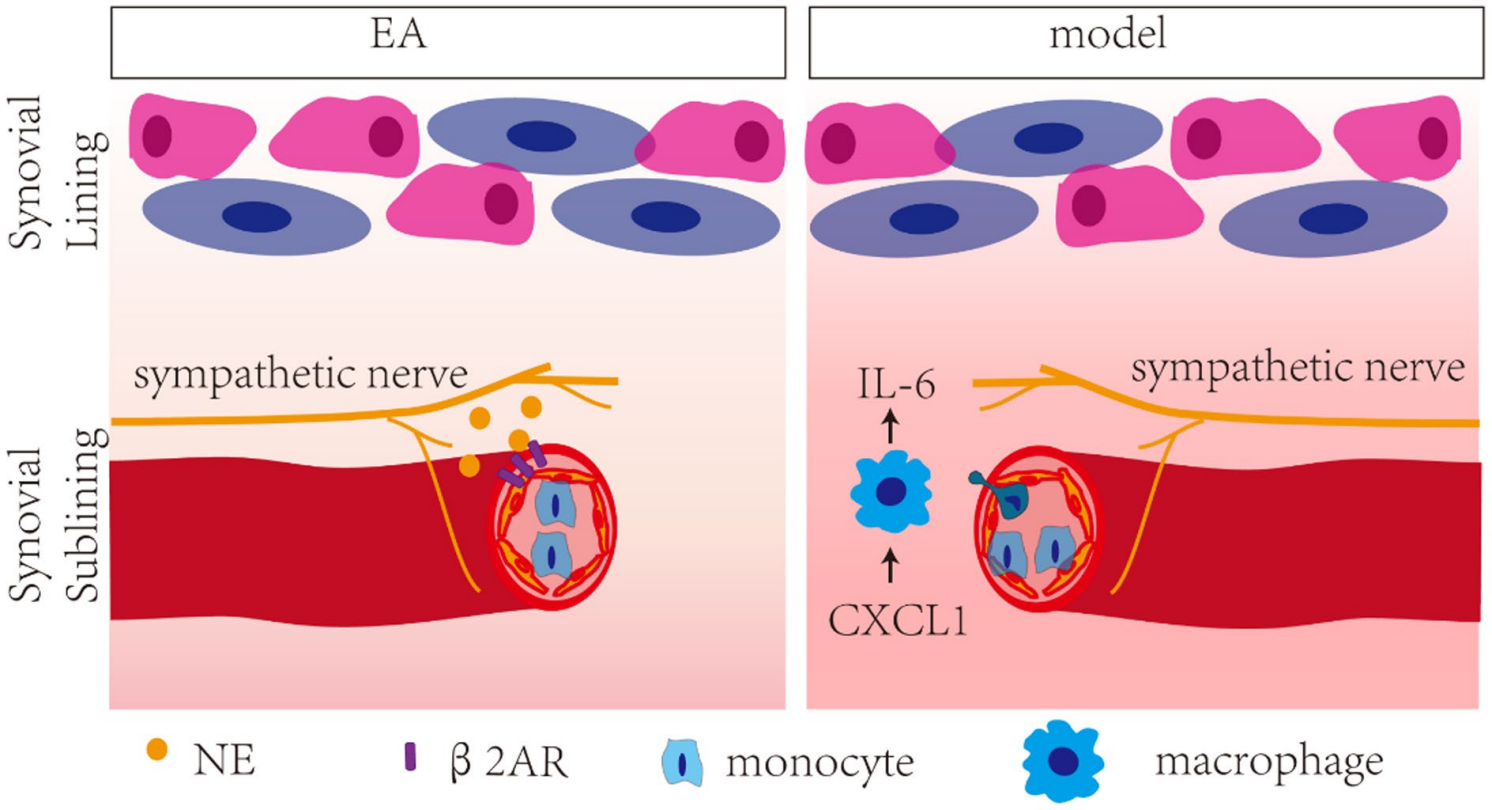
Schech map of the anti-inflammatory effect of EA in MIA-induced KOA rats. EA inhibited CXCL1/CXCR2-dependent recruitment of macrophage and its overexpression of IL-6 in the synovium by activating the β2-AR.

In the present study, a positive correlation was observed between the levels of CXCL1 and IL-6, and antagonists of NE (6-OHDA) and β2-AR (ICI 118,551) blocked the inhibition effect of EA on CXCL and IL-6, indicating that EA-induced increase in NE inhibited CXCL1-dependent overexpression of IL-6 in the synovium by activating β2-AR. Chemokines are well-recognized for their involvement in the migration of circulating cells into inflammatory tissue. CXCL1 acts as an important proinflammatory chemokine by binding specifically to its corresponding G protein-coupled receptor chemokine (CXC motif) receptor 2 (CXCR2) (Hou et al., 2020). CXCL1 levels are low under normal physiological conditions and substantially increase during inflammatory conditions, and CXCL1 expression appears to be increased in OA patients (Borzi et al., 1999). CXCR2 activates multiple signaling pathways, such as the PI3K/Akt, PLC/PKC, MAPK, ERK, P38, and JAK/STAT3 pathways (Cheng et al., 1871; Zhang et al., 2017). MAPK proteins are a family of serine/threonine protein kinases commonly activated by proinflammatory cytokine stimulation, which in turn regulate the production of inflammation mediators (Kaminska, 2005). The chemokine CXCL1 induced the overexpression of cytokine IL-6 in the synovium of KOA patients through the CXCR2, c-Raf, MAPK, and AP-1 pathways (Hou et al., 2020). Our findings indicate that CXCL1/CXCR2 axis triggers the activation of MAPK signaling, including MEK and ERK proteins to promote synovitis, which was inhibited by EA. Moreover, EA reversed the increased expression of the CXCR2 in macrophages. CXCL1 is the master chemokine responsible for the recruitment of macrophages by binding with CXCR2 (Xu et al., 2021). Overall, our study demonstrated that EA inhibited CXCL1-CXCR2-dependent recruitment of macrophage and its overexpression of IL-6 in the synovium by activating the sympathetic noradrenergic signaling pathway (Figure 9).

Several approaches have been developed to address the relationship between central and peripheral neuroinflammation with LC (Feinstein et al., 2016). Central activation of the LC-NE system leads to the central release of NE throughout the brain, thus inducing the peripherally increasing sympathetic output, which includes the release of NE from the varicose sympathetic nerve terminals and epinephrine from the adrenal medulla. Functionally, two sympathy-excitatory brain areas—the PVN/CRH and LC/NE/sympathetic systems—seem to participate in a positive, reverberatory feedback loop (Elenkov et al., 2000). However, in the present study, no c-fos + CRH positive neuron was observed in the PVN, and no obvious change was observed in the plasma levels of epinephrine and CORT, but increased synovial NE levels were observed after EA. The results suggested that the integrity of the LC descending pathway is crucial for controlling arthritic joint inflammation by EA, consistent with that of a previous study on the anti-inflammatory role of the LC descending pathway in experimental arthritis (Bassi et al., 2017).

Low-level inflammation is believed to contribute to degenerative changes and the development of peripheral sensitization and nociceptive pain. Pro-inflammatory cytokines such as IL-6 and TNF-α have been demonstrated to induce the peripheral sensitization of joint nociceptors and contribute to enhanced pain sensation (Schaible, 2014). A significant correlation between KOA pain and CXCL1, IL-6 has also been reported (Nees et al., 2019; Valiate et al., 2019). The attenuation of early-phase inflammation by cannabidiol has been confirmed to prevent pain and nerve damage in rat OA (Philpott et al., 2017). In the present study, EA suppressed the inflammation in the synovium on day 9 after MIA and alleviated referred hind aw pain on day 14 after MIA. Therefore, EA attenuated the mid-term phase of inflammation to prevent referred pain in the later phase in KOA rats, yet its mechanism requires further investigation.

Overall, our study identifies the effect of EA on the anti-inflammatory effect in KOA rats and uncovers the mechanism related to sympathetic noradrenergic signaling. Our study also helps in the development of novel therapeutic strategies for inflammatory diseases.

## 5. Conclusion

EA at the midterm phase of inflammatory pain activated the sympathetic transmitter NE and its β2-AR in the synovium without increasing the systemic NE or adrenaline in the circulation, to inhibit CXCL1-CXCR2-dependent overexpression of IL-6 in the synovial macrophages on day 9 after MIA injection and to prevent referred hind paw pain on day 14 after MIA injection, thereby exerting a local effect on the inflammatory pain in MIA-induced KOA rats.

## Data availability statement

The datasets presented in this study can be found in online repositories. The names of the repository and accession number can be found at: https://www.iprox.cn/, IPX0005493000.

## Ethics statement

The animal study was reviewed and approved by Institutional Animal Welfare and Use Committee of the institute of Acupuncture and Moxibustion, China Academy of Chinese Medical Sciences.

## Author contributions

WH: conceptualization and project administration. WC, X-NZ, WH, Y-SS, X-YW, H-CL, Y-HL, H-YW, and Z-YQ: methodology. X-NZ and WC: software and data curation. WH, X-NZ, and WC: writing—original draft preparation. WH and X-HJ: writing—review and editing. All authors have read and agreed to the published version of the manuscript.

## Funding

This work was supported by grants from the National Key R&D Program of China (No. 2019YFC1709002), CACMS Innovation Fund (No. CI2021A03404), National Natural Science Foundation of China (No. 82130122), and Beijing National Science Foundation (No. 7222289).

## Conflict of interest

The authors declare that the research was conducted in the absence of any commercial or financial relationships that could be construed as a potential conflict of interest.

## Publisher’s note

All claims expressed in this article are solely those of the authors and do not necessarily represent those of their affiliated organizations, or those of the publisher, the editors and the reviewers. Any product that may be evaluated in this article, or claim that may be made by its manufacturer, is not guaranteed or endorsed by the publisher.

## Footnotes

^1^ https://www.omicsolution.com/wkomics/main/

## Abbreviations

EA: Electroacupuncture
MIA: Sodium monoiodoacetate
KOA: Knee osteoarthritis
TNF-α: Tumor necrosis factor
IL-1β: Interleukin-1β.
